# Zinc Oxide Nanoparticles Treatment Maintains the Postharvest Quality of Litchi Fruit by Inducing Antioxidant Capacity

**DOI:** 10.3390/foods13213357

**Published:** 2024-10-23

**Authors:** Xiaomeng Guo, Qiao Li, Tao Luo, Dandan Xu, Difa Zhu, Jingyi Li, Dongmei Han, Zhenxian Wu

**Affiliations:** 1 Guangxi Key Laboratory of Health Care Food Science and Technology, School of Food and Biological Engineering, Hezhou University, Hezhou 542899, China; guoxm_scau@163.com; 2 Guangdong Provincial Key Laboratory of Postharvest Science of Fruits and Vegetables, Engineering Research Center of Southern Horticultural Products Preservation, Ministry of Education, College of Horticulture, South China Agricultural University, Guangzhou 510642, China; liqiao@stu.scau.edu.cn (Q.L.); luotao0502@scau.edu.cn (T.L.); dfzhu@stu.scau.edu.cn (D.Z.); jyli0520@163.com (J.L.); 3 School of Food Science and Engineering, Hainan University, Haikou 570228, China; happyxudandan@126.com; 4 Key Laboratory of South Subtropical Fruit Biology and Genetic Resource Utilization, Ministry of Agriculture and Rural Affairs, Guangdong Provincial Key Laboratory of Science and Technology Research on Fruit Tree, Institute of Fruit Tree Research, Guangdong Academy of Agricultural Sciences, Guangzhou 510640, China; handongmei@gdaas.cn

**Keywords:** litchi, postharvest, quality, zinc oxide nanoparticles (ZnO NPs), antioxidant, pericarp browning, decay

## Abstract

Pericarp browning and fruit decay severely reduce the postharvest quality of litchi. Improving the antioxidant capacity of the fruit is an effective way to solve these problems. In our study, the appropriate zinc oxide nanoparticles (ZnO NPs) treatment and its mechanism of action on the storability of litchi was investigated. Litchi fruit was soaked in a 100 mg·L^−1^ ZnO NPs suspension, water, and 500 mg·L^−1^ prochloraz for 2 min, respectively. The results showed that the ZnO NPs treatment delayed pericarp browning and decay in litchi fruit and was more effective than prochloraz treatment. The ZnO NPs-treated fruit showed significantly increased contents of total anthocyanin, total phenols, and activities of DPPH scavenging, superoxide dismutase, and glutathione peroxidase, as well as the lowest activities of polyphenol oxidase and laccase. ZnO NPs generated hydrogen peroxide and superoxide anion radicals, which were beneficial in slowing down the decay and inducing antioxidant capacity. However, these reactive oxygen species also consumed catalase, peroxidase, glutathione, and glutathione peroxidase. This means that litchi should be treated with an appropriate concentration of ZnO NPs. We concluded that treatment with a 100 mg·L^−1^ ZnO NPs suspension could induce antioxidant capacity, which is a promising and effective method to maintain the postharvest quality of litchi.

## 1. Introduction

Litchi (*Litchi chinensis* Sonn.) is a subtropical to tropical fruit native to China [1]. Litchi fruit has a high commercial value due to its attractive color, unique flavor, and rich nutritional content [2]. After harvest, litchi fruit is susceptible to pericarp browning and fruit decay, which significantly reduces its commodity value and shelf life [3]. Prochloraz treatment is a common postharvest application on litchi fruit and could effectively control pericarp browning and decay caused by disease [3,4,5]. However, pathogen resistance and human health risks from fungicides cannot be ignored. As a result, more methods of preserving litchi fruit are being explored to reduce or replace the use of fungicides.

Studies have shown that treatment with some substances that affect antioxidant-related indicators in litchi fruit can delay pericarp browning, decay, and senescence, and improve stress resistance. Some substances have antioxidant activity on their own, such as vanillin–taurine Schiff base compound [6], polyphenol [7], hydrogen water [8], hydrogen sulfide [9,10], *α*-Lipoic acid [11], apple polyphenols [12,13], *aloe vera* gel [14], chitosan [15], methionine [16], tea polyphenols [17], pyrogallol [18], and anthocyanin extract from black bean seed coat [19], while others induce litchi to increase its antioxidant capacity, such as GR24 [20], 6-pentyl-2*H*-pyran-2-one [21], melatonin [22,23,24,25], methyl jasmonate [26,27], oxalic acid [28,29], and nitric oxide [30].

Nanotechnology offers a number of new approaches to fruit preservation [31,32]. Many types of nanoparticles have been proven to act as antimicrobials and to induce oxidative stress in plants [33,34]. Inorganic nanoparticles include mainly zinc oxide, silver, iron oxide, titanium dioxide, and silicon dioxide [32]. Among them, zinc oxide nanoparticles (ZnO NPs) have been widely used as antimicrobial and antioxidant agents due to their high surface-to-volume ratio, generation of reactive oxygen species (ROS), and release of Zn^2+^ ions [35,36,37,38], leading to their use in various applications, including food packaging [31,36,39]. It is worth noting that the positive effects of ZnO NPs in food packaging on the postharvest quality of fruit have been extensively documented. For example, composites of chitosan oligosaccharide and ZnO NPs for tomato application [40]; cassava starch-based multifunctional coating with ZnO NPs for passion fruit application [41]; gelatin film containing red roselle extract, ZnO NPs and Litsea cubeba oil Pickering emulsion, or alginate-based ZnO NPs coating for mango application [42]; chitosan–ZnO NPs coating supplemented with bergamot essential oil for table grape application [43,44]; and chitosan coatings modified with ZnO NPs for strawberry application [45]. Studies have analyzed the role of ZnO NPs treatment in controlling postharvest diseases and their mechanism of action [36,46,47,48]. However, the action mechanism of ZnO NPs treatment on postharvest fruit needs to be studied in detail. Therefore, the action mechanism of ZnO NPs on litchi fruit was investigated, particularly the role of ZnO NPs treatment in improving the antioxidant capacity of litchi fruit in this study.

## 2. Materials and Methods

### 2.1. Plant Material and Treatments

Litchi fruit (cultivar ‘Jingganghongnuo’) at commercial maturity (bright red pericarp) was obtained from Guangzhou, China. More than 800 good-quality fruit were selected and separated into three groups. Each of the three groups of fruit was soaked in water (negative control), 500 mg·L^−1^ prochloraz (Jiangsu Huifeng Bio Agriculture Co., Ltd., Huaian, China) (positive control), and a 100 mg·L^−1^ ZnO NPs suspension, containing ZnO NPs with an average diameter of 20 nm (Guangzhou Metallurgy Industry Co., Ltd., Guangzhou, China), for 2 min. The ZnO NPs suspension was prepared with the water containing 0.3 mL·L^−1^ Tween 80 (Macklin Biochemical Technology Co., Ltd., Shanghai, China); therefore, 0.3 mL·L^−1^ Tween 80 was also added to the water and prochloraz treatments. After air drying, approximately 20 fruits were placed in a polyethylene terephthalate tray and wrapped in 0.01-mm polyvinyl chloride film. More than 40 trays of fruit from the three treatments were stored at 25 ± 2 °C. Three trays of fruit on days 0, 2, and 4, and five trays of fruit on day 6 were randomly selected for observation and sampling for each treatment. The pericarp for sampling was ground in liquid nitrogen and stored at −80 °C for subsequent determinations.

### 2.2. Evaluation of Pericarp Browning Index and Decay

The pericarp browning index and decay were evaluated with the method described by Guo et al. [49].

### 2.3. Measurement of Secondary Metabolite-Related Indices

The contents of total anthocyanin, phenols, and flavonoids were measured with the methods described by Shuai et al. and Zhang et al. [13,50]. The polyphenol oxidase (PPO) activity was measured with the method of Kumar et al. [51], with slight modifications. The laccase (Lac) activity was measured with the method of Fang et al. and Zhang et al. [52,53], with slight modifications. The details of the determination of PPO and Lac activities are shown in the “Supplementary Materials”.

### 2.4. Measurement of Reactive Oxygen Species (ROS)-Related Indices

The superoxide anion radical (O_2_^−·^) production rate and 2,2-diphenyl-1-picrylhydrazyl (DPPH) scavenging activity were measured with the methods described by Zhang et al. [13]. The hydrogen peroxide (H_2_O_2_) content was measured with the method of Patterson et al. [54], with slight modifications. The activities of superoxide dismutase (SOD), catalase (CAT), and peroxidase (POD) were measured with the methods of Toivonen and Sweeney, Chance and Maehly, and Jing et al. [18,55,56], with slight modifications. The details of the determination of the H_2_O_2_ content and the activities of SOD, CAT, and POD are shown in the “Supplementary Materials”.

### 2.5. Measurement of GSH-Related Indices

The contents of glutathione (GSH) and glutathione disulfide (GSSG) were measured with the methods of Rahman et al. and Su et al. [12,57], with slight modifications. The GPX was measured with the method of Hafeman et al. and Monteiro et al. [58,59], with slight modifications. The GR was measured with the method of Smith et al. [60], with slight modifications. The details are shown in the “Supplementary Materials”.

### 2.6. Measurement of Protein Content

Protein content was measured with the method described by Bradford [61]. The activities of PPO, Lac, SOD, CAT, POD, GPX, and GR were expressed as U·mg^−1^ protein.

### 2.7. Statistical Analysis

One-way ANOVA and Duncan’s multiple range test were performed to analyze the differences between the means of the different treatments at the same sampling time. The Bivariate Pearson correlation was used to test whether there was a statistically significant linear relationship between two measured indices. For hierarchical clustering, the Euclidean distance algorithm was chosen for the similarity measure and the complete linkage clustering algorithm for the clustering.

## 3. Results

### 3.1. Effect of ZnO NPs Treatment on Pericarp Browning and Decay of Litchi Fruit

**Figure 1:**
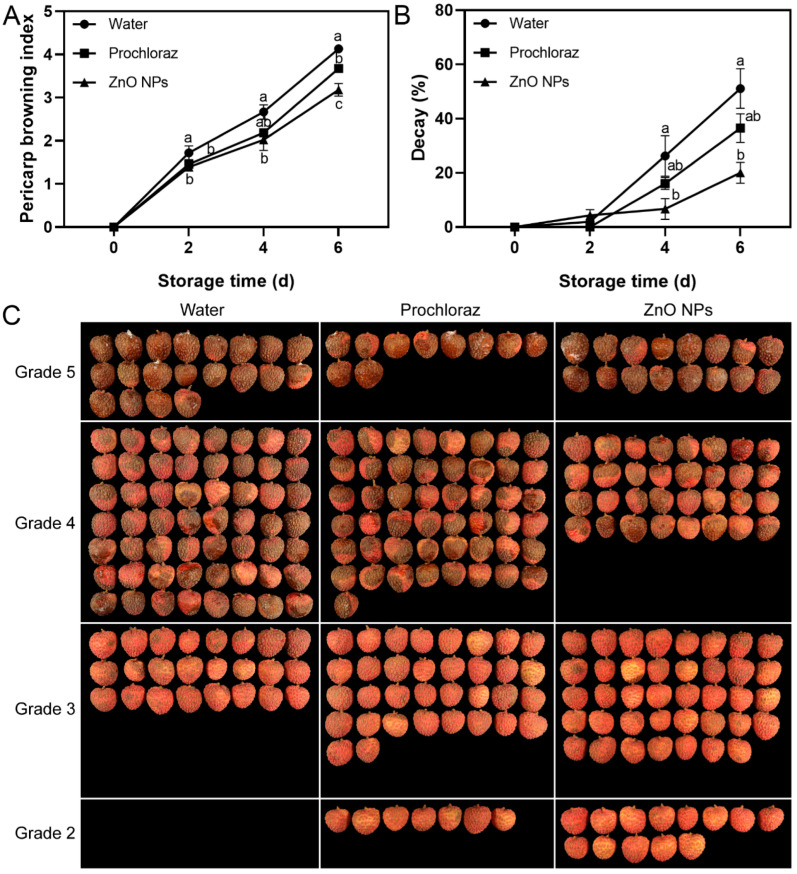
Effects of ZnO NPs treatment on litchi pericarp browning index (**A**) and decay (**B**) during storage time. (**C**) shows the appearance of treated fruit according to the grade of pericarp browning on day 6. Data represent the mean of three replicates ± standard error of the mean (SEM). Different small letters represent significant differences between treatments for each sampling time at *p* < 0.05, while data without small letters are not significantly different.

The pericarp browning index of ZnO NPs-treated fruit was significantly lower than that of the water-treated fruit at 2–6 d and significantly lower than that of the prochloraz-treated fruit at 6 d (Figure 1A). The ZnO NPs treatment maintained the pericarp browning grades of more fruit at grades 2 and 3 on day 6 (Figure 1C). The decay of ZnO NPs-treated fruit was significantly lower than that of the water-treated fruit at 4–6 d. There was no significant difference between the decay of ZnO NPs-treated and prochloraz-treated fruit during storage (Figure 1B). These results indicated that treatment with ZnO NPs effectively inhibited pericarp browning and the decay of litchi fruit and was more effective than the prochloraz treatments.

### 3.2. Effect of ZnO NPs Treatment on Secondary Metabolite-Related Indices of Litchi Fruit

**Figure 2:**
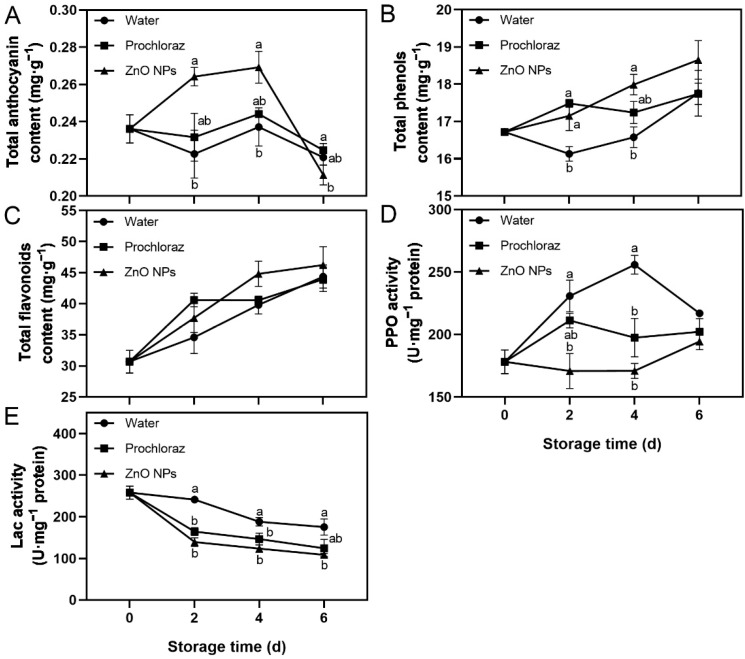
Effects of ZnO NPs treatment on total anthocyanin content (**A**), total phenols content (**B**), total flavonoids content (**C**), polyphenol oxidase (PPO) activity (**D**), and laccase (Lac) activity (**E**) of litchi fruit during storage time. Data represent the mean of three replicates ± SEM. Different small letters represent significant differences between treatments for each sampling time at *p* < 0.05, while data without small letters are not significantly different.

Fruit treated with ZnO NPs showed an increase in total anthocyanin content from 0–4 d, followed by a rapid decrease after 4 d. In addition, total phenols content, total flavonoids content, and PPO activity increased, while Lac activity decreased throughout storage (Figure 2). On days 2 and 4, the total anthocyanin content of ZnO NPs-treated fruit was significantly higher than that of water-treated fruit, and not significantly different from that of prochloraz-treated fruit. On day 6, however, it was significantly lower than in prochloraz-treated fruit (Figure 2A). The total phenols content of ZnO NPs-treated fruit was significantly higher than that of water-treated fruit on days 2 and 4 (Figure 2B). On the contrary, the PPO activity of ZnO NPs-treated fruit was significantly lower than that of water-treated fruit on days 2 and 4 (Figure 2D). During storage, the Lac activity of ZnO NP-treated fruit was always significantly lower than that of water-treated fruit (Figure 2E). Furthermore, there was no significant difference in the total flavonoids content between treatments. The total phenols content, PPO activity, and Lac activity of ZnO NPs-treated fruit were not significantly different from those of prochloraz-treated fruit (Figure 2B–E). These results indicated that treatment with ZnO NPs increased the levels of secondary metabolites and inhibited the activities of secondary metabolite-related enzymes in litchi pericarp. The effect of ZnO NPs treatment on secondary metabolite-related indices was similar to that of prochloraz treatment and superior to that of water treatment.

### 3.3. Effect of ZnO NPs Treatment on ROS-Related Indices of Litchi Fruit

**Figure 3:**
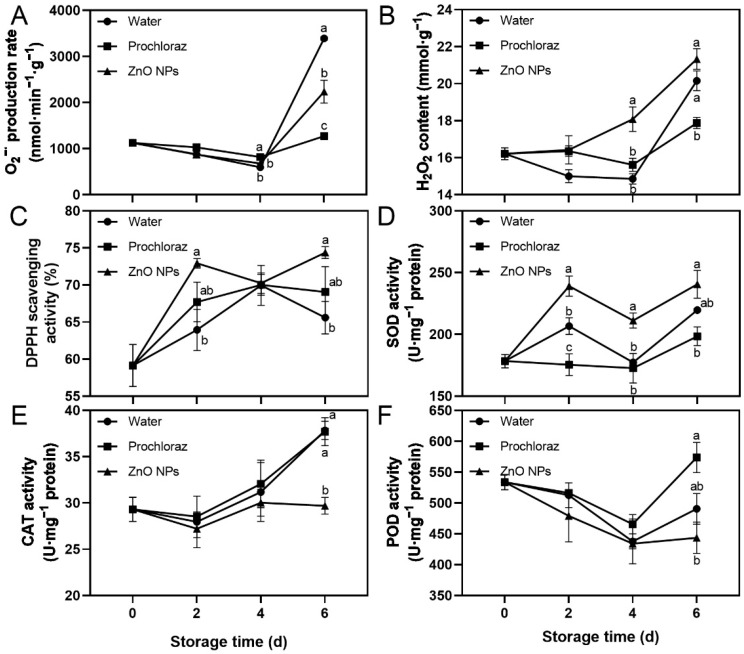
Effects of ZnO NPs treatment on superoxide anion radical (O_2_^−·^) production rate (**A**), hydrogen peroxide (H_2_O_2_) content (**B**), DPPH scavenging activity (**C**), superoxide dismutase (SOD) activity (**D**), catalase (CAT) activity (**E**), and peroxidase (POD) activity (**F**) of litchi fruit during storage time. Data represent the mean of three replicates ± SEM. Different small letters represent significant differences between treatments for each sampling time at *p* < 0.05, while data without small letters are not significantly different.

The O_2_^−·^ production rate, H_2_O_2_ content, DPPH scavenging activity, and SOD activity of ZnO NPs-treated fruit showed an increasing trend, while POD activity decreased (Figure 3). On day 6, the O_2_^−·^ production rate of ZnO NPs-treated fruit was significantly lower than that of water-treated fruit, and significantly higher than that of prochloraz-treated fruit (Figure 3A). The H_2_O_2_ content of ZnO NPs-treated fruit was significantly higher than that of water-treated fruit at 4 d, and significantly higher than that of prochloraz-treated fruit at 4 d and 6 d (Figure 3B). The CAT activity of ZnO NPs-treated fruit was significantly lower than that of water- and prochloraz-treated fruit on day 6 (Figure 3E). The DPPH scavenging activity of ZnO NPs-treated fruit was significantly higher than that of water-treated fruit on days 2 and 6, and not significantly different from that of prochloraz-treated fruit during storage (Figure 3C). The SOD activity of ZnO NPs-treated fruit was significantly higher than that of water-treated fruit on days 2 and 4, and significantly higher than that of prochloraz-treated fruit during storage (Figure 3D). The POD activity of ZnO NPs-treated fruit was significantly lower than that of prochloraz-treated fruit on day 6, and not significantly different from that of water-treated fruit during storage (Figure 3F). These results indicated that ZnO NPs treatment promoted the generation of ROS. Moreover, ZnO NPs treatment also contributed to some extent to the antioxidant activity of litchi pericarp.

### 3.4. Effect of ZnO NPs Treatment on GSH-Related Indices of Litchi Fruit

**Figure 4:**
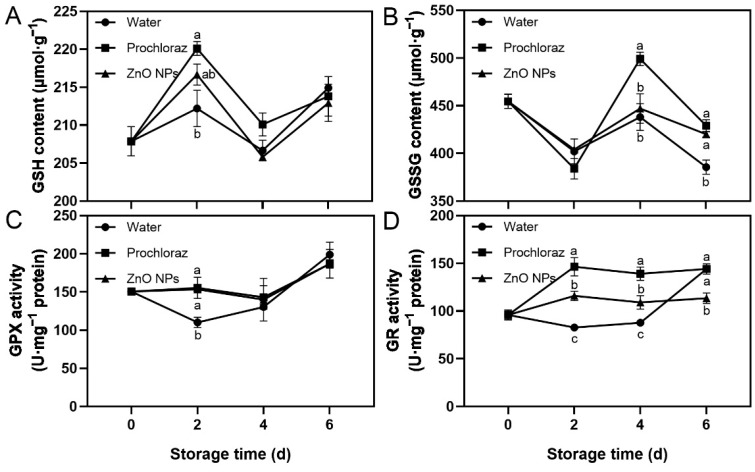
Effects of ZnO NPs treatment on glutathione (GSH) content (**A**), glutathione disulfide (GSSG) content (**B**), glutathione peroxidase (GPX) activity (**C**), and glutathione reductase (GR) activity (**D**) of litchi fruit during storage time. Data represent the mean of three replicates ± SEM. Different small letters represent significant differences between treatments for each sampling time at *p* < 0.05, while data without small letters are not significantly different.

The trend of GSH content in ZnO NPs-treated fruit during storage was increasing, then decreasing, followed by increasing again, while the trend of GSSG content was slightly decreasing. The GPX activity of ZnO NPs-treated fruit was increased (Figure 4). The GSH content and GPX activity of ZnO NPs-treated and prochloraz-treated fruit were significantly higher than those of water-treated fruit on day 2 (Figure 4A,C). The GSSG content of ZnO NPs-treated and water-treated fruit was significantly lower than that of prochloraz-treated fruit on day 4, and ZnO NPs-treated and prochloraz-treated fruit was significantly higher than that of water-treated fruit on day 6 (Figure 4B). The GR activity of ZnO NPs-treated fruit was significantly lower than that of prochloraz-treated fruit during storage, and significantly higher than that of water-treated fruit on days 2 and 4 (Figure 4D). These results indicated that treatment with ZnO NPs increased GSH content and GPX activity, but prochloraz treatment had a greater effect on GSH-related indices.

### 3.5. Correlation and Hierarchical Clustering of Pericarp Browning and Fruit Decay with Antioxidant Indices

**Figure 5:**
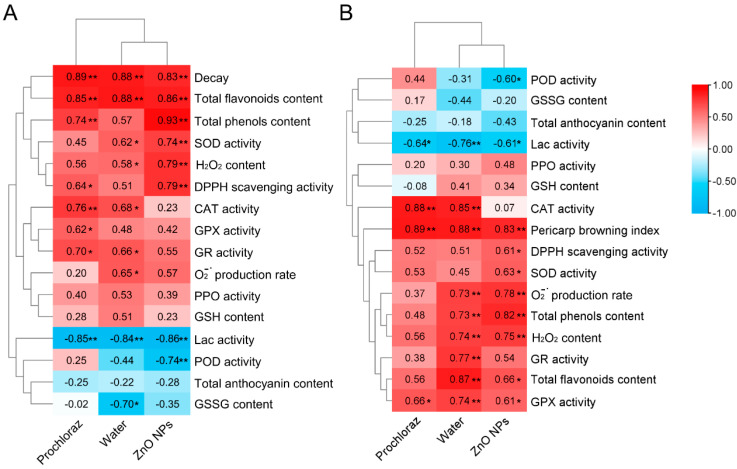
Correlation and hierarchical clustering of pericarp browning with fruit decay and antioxidant indices (**A**), and correlation and hierarchical clustering of fruit decay with pericarp browning and antioxidant indices (**B**). *, significant correlation (*p* < 0.05); **, significant correlation (*p* < 0.01).

Pairwise correlation analysis was performed to identify the correlation between pericarp browning and antioxidant indices, as well as fruit decay and antioxidant indices of litchi fruit after ZnO NPs, water, and prochloraz treatments (Figure 5). In ZnO NPs-treated fruit, pericarp browning was significantly positively correlated with decay, total flavonoids content, total phenols content, SOD activity, H_2_O_2_ content, and DPPH scavenging activity, but significantly negatively correlated with Lac activity and POD activity; decay was significantly positively correlated with pericarp browning index, DPPH scavenging activity, SOD activity, O_2_^−·^ production rate, total phenols content, H_2_O_2_ content, total flavonoids content, and GPX activity, but significantly negatively correlated with POD activity and Lac activity. Correlation analysis of the water-treated fruit showed that both pericarp browning and decay were correlated with most of the antioxidant indices. Compared to prochloraz-treated fruit, pericarp browning and decay of ZnO NPs-treated fruit had a higher correlation with antioxidant indices.

## 4. Discussion

### 4.1. ZnO NPs Treatment Delayed Pericarp Browning and Fruit Decay by Inducing Antioxidant Capacity of Litchi Fruit

In our study, pericarp browning and fruit decay are major factors affecting the postharvest quality of litchi. Treatment with a 100 mg·L^−1^ ZnO NPs suspension delayed pericarp browning and fruit decay, indicating that ZnO NPs treatment is an effective method for maintaining the postharvest quality of litchi fruit (Figure 1). Postharvest application in orange [62], strawberry [63,64], and pomegranate [65] suggests that treatment with ZnO NPs improves the antioxidant capacity of the fruit. Interestingly, our study showed similar results.

Total anthocyanin, total phenols, and total flavonoids are secondary metabolites with antioxidant activity, as well as metabolites closely related to the color of the litchi pericarp [3,66]. PPO and Lac are enzymes associated with pericarp browning [52,67]. In the fruit treated with ZnO NPs, the contents of total anthocyanin and total phenols increased considerably. The total flavonoids content also increased, but did not reach a significant level, and the activities of PPO and Lac were effectively inhibited (Figure 2). It is worth noting that the induction of secondary metabolite-related indices by the ZnO NPs treatment was more pronounced on days 2 and 4 (Figure 2). Similarly, pomegranate coated with carboxymethyl cellulose and 0.2% ZnO NPs, strawberries coated with 1.5% sodium alginate and 1.25 g/L ZnO NPs, and strawberries treated with ZnO NPs also showed higher levels of total phenols and anthocyanin [63,64,65]. In addition, orange coated with 0.5% chitosan and 0.50 g/L ZnO NPs had lower PPO activity [62].

The excessive accumulation of ROS leads to membrane lipid peroxidation, which causes damage to fruit membranes. ROS mainly include H_2_O_2_, O_2_^−·^, and ·OH, while several enzymes can scavenge them, such as SOD, CAT, and POD. DPPH scavenging activity is used to assess the total non-enzymatic antioxidant activity [8,13]. In the fruit treated with ZnO NPs, DPPH scavenging activity and SOD activity upregulated substantially. However, the H_2_O_2_ content and O_2_^−·^ production rate were higher, and the activities of CAT and POD were lower (Figure 3). We believed that this was because ZnO NPs produce H_2_O_2_ and O_2_^−·^, especially a large amount of H_2_O_2_ [68,69]. Therefore, CAT and POD were used to scavenge H_2_O_2_ [70]. Similar to results in other studies, the orange coated with 0.5% chitosan and 0.50 g/L ZnO NPs had lower POD activity, and the strawberry coated with 1.5% sodium alginate and 1.25 g/L ZnO NPs had lower POD activity and higher SOD activity [62,63].

GSH plays an important role in intracellular ROS defense in plants. GSSG is the oxidized form of GSH. GPX catalyzes the conversion of GSH to GSSG, while GSSG can be reduced by GR to regenerate GSH. Both GSH and GSSG directly or indirectly remove excess ROS and their reaction products. Additionally, the balance between them is crucial for maintaining the cellular redox state [71,72]. In the fruit treated with ZnO NPs, the trends of GSH content and GPX activity were upward, but the GSSG content and GR activity were not obviously changed. We thought that treatment with ZnO NPs induced GSH production and increased GPX activity, which facilitated the conversion of GSH to GSSG. However, most of the GSH and GPX could be used to scavenge ROS [72].

Correlation analysis showed that pericarp browning and fruit decay were correlated with the antioxidant system of litchi fruit. ZnO NPs are oxide nanoparticles, therefore pericarp browning and fruit decay of ZnO NPs-treated fruit were significantly correlated with many antioxidant-related indices. However, pericarp browning and fruit decay in prochloraz-treated fruit did not appear to be significantly related to the antioxidant system (Figure 5). These results also demonstrated the different mechanisms by which prochloraz and ZnO NPs maintain fruit quality. Prochloraz is a broad-spectrum imidazole fungicide that acts as an antimicrobial by inhibiting ergosterol biosynthesis in fungi [73]. As a result, prochloraz treatment killed pathogens directly and delayed the decay to maintain fruit quality, while ZnO NPs exert antimicrobial activity through physical contact between ZnO NPs and the bacterial cell wall, ROS generation, and Zn^2+^ ions release [35]. Meanwhile, we found that the antioxidant capacity of the fruit was induced (Figure 2, Figure 3 and Figure 4). Therefore, ZnO NPs treatment delayed decay and pericarp browning and improved stress resistance to maintain fruit quality.

### 4.2. Applications and Challenges of ZnO NPs Treatment

Although appropriate treatment with ZnO NPs could maintain fruit quality, high concentrations of ZnO NPs could have some negative effects. Previous studies have shown that the activities of antioxidant enzymes in plants increased at lower concentrations of nanoparticles and decreased at higher concentrations of nanoparticles. This is because lower concentrations of nanoparticles increase the antioxidant capacity of the plant to scavenge ROS, resulting in a balance between ROS production and scavenging, but higher concentrations of nanoparticles cause an oxidative burst in the plant and the plant is unable to combat ROS production [74]. The effect of nanoparticles on antioxidant capacity may be related to differences in plant species, type and size of nanoparticles, concentration and duration of treatment, and experimental conditions. As nanoparticles, ZnO NPs produce ROS, which are beneficial for antibacterial activity and activate the plant’s antioxidant capacity [34,35]. However, excessive ROS can also cause damage to biomolecules and organelle structures [75]. For example, treatment with 0.5% ZnO NPs was most effective in maintaining quality and controlling decay in strawberries, but the fruit was less luminous [64]. In our study, the fruit treated with ZnO NPs significantly inhibited pericarp browning and decay, significantly increased the antioxidant-related indices including total anthocyanin content, total phenols content, DPPH scavenging activity, SOD activity, and GPX activity. However, the treatment exhibited a sharp decrease in total anthocyanin content on day 6. Additionally, large amounts of H_2_O_2_ and O_2_^−·^ were produced and CAT, POD, GSH, and GPX could be consumed during storage (Figure 2, Figure 3 and Figure 4). Therefore, the concentration of ZnO NPs treatment used is very important. Through the preliminary experiments of 25, 50, 100, and 200 mg·L^−1^ ZnO NPs suspensions, we found that the optimal concentration of ZnO NPs for litchi treatment was 100 mg·L^−1^. Higher concentrations of ZnO NPs treatment instead accelerate pericarp browning. If the ZnO NPs are formulated in combination with other substances, the influence of the other substances on the adhesion of the ZnO NPs should also be considered. 

In conclusion, we claimed that a 100 mg·L^−1^ ZnO NPs suspension treatment is an effective method to maintain the postharvest quality of litchi fruit and is expected to be an alternative to fungicide treatment. In the future, the application of ZnO NPs in combination with other substances could be considered to improve the preservation effect and reduce its negative effects. Moreover, this study investigated the action mechanism of ZnO NPs from the perspective of the litchi fruit, which provided a reference for the application of ZnO NPs treatment for the preservation of other fruit and vegetables.

## 5. Conclusions

Treatment with a 100 mg·L^−1^ ZnO NPs suspension delayed the pericarp browning and fruit decay of litchi. Antioxidant-related indices, such as total anthocyanin content, total phenols content, DPPH scavenging activity, SOD activity, and GPX activity, were considerably increased. The activities of enzymes closely related to pericarp browning, PPO, and Lac, were significantly inhibited. Therefore, treatment with a 100 mg·L^−1^ ZnO NPs suspension effectively induced the antioxidant capacity and maintained the postharvest quality of litchi fruit. This study also provides a reference for further application of ZnO NPs treatment in the field of postharvest. 

## Data Availability

The original contributions presented in the study are included in the article/Supplementary Material, further inquiries can be directed to the corresponding author.

## Supplementary Materials

The following supporting information can be downloaded at: https://www.mdpi.com/article/10.3390/foods13213357/s1. Details of the methods.
