# Supplementary material for: Zinc Oxide Nanoparticles Treatment Maintains the Postharvest Quality of Litchi Fruit by Inducing Antioxidant Capacity
Source: Foods. 2024 Oct 23;13(21):3357. doi: 10.3390/foods13213357 (PMC11545015; doi:10.3390/foods13213357)
Supplement: Supplementary file 1 [file foods-13-03357-s001.zip › foods-3214561-supplementary.pdf]

Zinc Oxide Nanoparticles Treatment Maintains the Postharvest Quality of Litchi Fruit by Inducing Antioxidant Capacity

Xiaomeng Guo [1,2], Qiao Li [2], Tao Luo [2], Dandan Xu [3], Difa Zhu [2], Jingyi Li [2], Dongmei Han [4] and Zhenxian Wu [2,*]

[1] Guangxi Key Laboratory of Health Care Food Science and Technology, School of Food and Biological Engineering, Hezhou University, Hezhou 542899, China; guoxm_scau@163.com

[2] Guangdong Provincial Key Laboratory of Postharvest Science of Fruits and Vegetables, Engineering Research Center of Southern Horticultural Products Preservation, Ministry of Education, College of Horticulture, South China Agricultural University, Guangzhou 510642, China; liqiao@stu.scau.edu.cn (Q.L.); luotao0502@scau.edu.cn (T.L.); dfzhu@stu.scau.edu.cn (D.Z.); jyli0520@163.com (J.L.)

[3] School of Food Science and Engineering, Hainan University, Haikou 570228, China; happyxudandan@126.com

[4] Key Laboratory of South Subtropical Fruit Biology and Genetic Resource Utilization, Ministry of Agriculture and Rural Affairs, Guangdong Provincial Key Laboratory of Science and Technology Research on Fruit Tree, Institute of Fruit Tree Research, Guangdong Academy of Agricultural Sciences, Guangzhou 510640, China; handongmei@gdaas.cn

[*] Correspondence: zhenxwu@scau.edu.cn; Tel.: +86-020-85280228

Supplementary Materials

The reagents used for the following measurement were purchased from the Macklin Biochemical Technology Co., Ltd., Shanghai, China.

1. Measurement of the activities of polyphenol oxidase (PPO), laccase (Lac) and peroxidase (POD)

Crude enzyme for the determination of PPO (EC 1.10.3.1), Lac (EC 1.10.3.2) and POD (EC 1.11.1.7) was extracted with 50 mmol·L^{-1} sodium phosphate buffer (pH 7.0) containing 5% (w/v) polyvinylpyrrolidone (PVP). The reaction solution for the determination of PPO activity contained crude enzyme and 100 mmol·L^{-1} catechol solution. One unit (U) of PPO activity was defined as the amount of enzyme that causes an increase in absorbance of 0.01 at 420 nm per minute. The reaction solution for the determination of Lac activity contained crude enzyme and 2 mmol·L^{-1} epicatechin solution. One U of Lac activity was defined as the amount of enzyme that causes an increase in absorbance of 0.1 at 380 nm per minute. The reaction solution for the determination of POD activity contained crude enzyme, 25 mmol·L^{-1} guaiacol solution, and 500 mmol·L^{-1} H_2O_2 solution. One U of POD activity was defined as the amount of enzyme that causes an increase in absorbance of 0.1 at 470 nm per minute.

2. Measurement of the hydrogen peroxide (H_2O_2) content and the activities of superoxide dismutase (SOD) and catalase (CAT)

H_2O_2 was extracted with cold acetone. After centrifugation, the supernatant was mixed with titanium tetrachloride-hydrochloric acid solution and concentrated ammonia solution. After centrifugation again, the precipitate was washed with cold acetone and dissolved in 2 mol·L^{-1} H_2SO_4. The absorbance was measured at 412 nm.

Crude enzyme for the determination of SOD (EC 1.15.1.1) and CAT (EC 1.11.1.6) was extracted with 100 mmol·L^{-1} sodium phosphate buffer (pH 7.5) containing 5% (w/v) PVP and 5 mmol·L^{-1} dithiothreitol (DTT). The reaction solution for the determination of SOD activity contained crude enzyme, 50 mmol·L^{-1} sodium phosphate buffer (pH 7.8), 130 mmol·L^{-1} L-methionine solution, 750 μmol·L^{-1} nitro-blue-tetrazolium (NBT) solution, 100 μmol·L^{-1} ethylenediaminetetraacetic acid disodium salt (EDTA-Na_2) solution, and 20 μmol·L^{-1} riboflavin solution. One U of SOD activity was defined as the amount of enzyme that causes a 50% inhibition of NBT reduction at 560 nm. The reaction solution for the determination of CAT activity contained crude enzyme and 20 mmol·L^{-1} H_2O_2 solution. One U of CAT activity was defined as the amount of enzyme that causes a decrease in absorbance of 0.01 at 240 nm per minute.

3. Measurement of the contents of glutathione (GSH) and glutathione disulfide (GSSG), and the activities of glutathione peroxidase (GPX) and glutathione reductase (GR)

GSH and GSSG were extracted with 50 g·L^{-1} trichloroacetic acid containing 5 mmol·L^{-1} EDTA-Na_2. The supernatant was used for the determination of GSH and GSSG content. The reaction solution for GSH determination contained the supernatant, 0.1 mol·L^{-1} sodium phosphate buffer (pH 7.5), and 4 mmol·L^{-1} 5,5′-Dithiobis- (2-nitrobenzoic acid) (DTNB) solution. The reaction solution was kept at 25°C for 10 min, and the absorbance was recorded at 412 nm. The supernatant used for GSSG determination was treated with 2-vinylpyridine at 25°C for 1 h to remove GSH. GSSG was converted to GSH by GR, and the amount of GSH was determined. The reaction solution contained the supernatant to remove GSH, 0.1 mol·L^{-1} sodium phosphate buffer (pH 7.5), 1 U GR, 4 mmol·L^{-1} NADPH, and 4 mmol·L^{-1} DTNB solution. GSH was then measured as described above.

Crude enzyme for the determination of GPX (EC 1.11.1.9) and GR (EC 1.8.1.7) were extracted with 100 mmol·L^{-1} sodium phosphate buffer (pH 7.5) containing 1 mmol·L^{-1} EDTA. The reaction solution for the determination of GPX activity contained crude enzyme, 1 mmol·L^{-1} GSH solution, and 1.5 mmol·L^{-1} H_2O_2. The reaction solution was kept at 37°C for 4 min, and then 1.67% metaphosphoric acid precipitation solution was added. After centrifugation, the supernatant was mixed with 0.32 mol·L^{-1} Na_2HPO_4 and 4 mmol·L^{-1} DTNB solution. The absorbance was recorded at 412 nm after 5 min. The control solution, containing boiled crude enzyme, was run at the same time as the samples. One U of GPX enzyme activity was defined as 1 μmol·L^{-1} GSH consumed per minute. The reaction solution for the determination of GR activity contained crude enzyme, enzyme extraction buffer, 5 mmol·L^{-1} GSSG solution, 4 mmol·L^{-1} NADPH solution, and 4 mmol·L^{-1} DTNB solution. One U of GR activity was defined as the amount of enzyme that causes a decrease in absorbance of 0.01 at 412 nm per minute.
